# Microbial transcriptome patterns highlight increased pedogenesis-related activity in arid soils under simulated humid conditions

**DOI:** 10.1186/s40793-025-00689-3

**Published:** 2025-03-17

**Authors:** Victoria Rodríguez, Alexander Bartholomäus, Susanne Liebner, Romulo Oses, Thomas Scholten, Dirk Wagner

**Affiliations:** 1 GFZ Helmholtz Centre for Geosciences, Section Geomicrobiology, Potsdam, Germany; 2 Institute of Biochemistry and Biology, University of Potsdam, Potsdam, Germany; 3 Centro Regional de Investigación y Desarrollo Sustentable de Atacama (CRIDESAT), Universidad de Atacama, Copiapó, Chile; 4 Department of Geosciences, Soil Science and Geomorphology, University of Tübingen, Tübingen, Germany; 5 Institute of Geosciences, University of Potsdam, Potsdam, Germany

**Keywords:** Metagenomic, Metatranscriptomic, Soil formation, Arid site, Semiarid site, Simulated humid climate, Chilean Coastal Cordillera

## Abstract

**Background:**

In arid and semiarid environments, microbial activity is restricted by low water availability and high evapotranspiration rates, and soil development is limited. Under humid conditions, such limitations can be overcome, accelerating pedogenesis by microbial processes. Our study aims to broaden our understanding of soil development under a climate change scenario toward humid conditions and to identify the microorganisms that help transform initial soils from arid and semiarid sites. We characterized pedogenetic microbial processes and how their gene expression differs between soils from arid and semiarid sites under a sixteen-week climate simulation experiment using metagenomic and metatranscriptomic approaches.

**Results:**

We found that an intense functional response is triggered under humid climate conditions in the arid site compared to the semiarid site, which showed greater resilience. The arid site undergoes higher transcription of genes involved in soil aggregate formation, phosphorus metabolism, and weathering, potentially adapting the development of arid sites to climate change. Additionally, a transcriptional reconfiguration linked to soil carbon and nitrogen dynamics suggests that soil microorganisms use available organic resources alongside autotrophy in response to increased moisture. *Pseudomonadota* and *Actinomycetota* dominated the overall transcriptional profile and specific functions associated with the early stages of soil development in both sites.

**Conclusions:**

Our findings highlight the rapid activation of pathways related to pedogenesis under humid conditions in arid sites, potentially driven by their metabolic requirements and environmental stressors, influencing soil development dynamics under global climate change.

**Supplementary Information:**

The online version contains supplementary material available at 10.1186/s40793-025-00689-3.

## Background

In arid and semiarid ecosystems, covering approximately 40% of the Earth’s terrestrial surface, soil development is conditioned by low precipitation and high potential evaporation rates [1]. Typical geomorphic processes in these environments include the formation of lamellar lakes and evaporite basins, wind-driven erosion, deposition of fine sands and silts, formation of sand drifts, dunes, and pediments, and physical weathering due to temperature gradients [2]. These processes and climate conditions impact soil formation factors, such as high levels of soluble salts, sodium ions adsorbed on organic matter and clay mineral surfaces, and the accumulation of gypsum, calcium, and silica, influencing plant growth and microbial development [3, 4]. Specifically, aridity challenges microbial activity by threatening microbial diversity and abundance [5], community composition [6], microbial interactions [7], resource availability, and enzymatic activity [8]. These threats feedback to soil development and influence the potential contributions of microorganisms to key soil formation processes, such as the stabilization of soil aggregates [9, 10], weathering of minerals/rocks [11], or organic matter formation [12].

Microbial communities in arid and semiarid environments exhibit genetic adaptations to cope with osmotic stress, high radiation exposure, and the absence of plant biomass, including traits associated with dehydration and radiation/desiccation tolerance [4]. Consequently, these dry environments are characterized by a prevalence of dormancy, sporulation, and osmoregulation genes, while genes related to nutrient cycling and complex organic compound degradation are less abundant [13–15]. Microbial communities in arid regions maintain a substantial capacity for carbon (C) fixation, phosphorous (P), and sulfur (S) cycling, while nitrogen (N) biogeochemistry is often limited [16–18]. Moreover, genes involved in N cycling and C-fixation (especially Calvin cycle and reductive tricarboxylic acid cycle-rTCA) are actively transcribed [19]. However, despite progress in understanding microbial adaptation to these environments, their role in soil formation and the differences between arid and semi-arid sites remain unclear. In particular, studies exploring the active contribution of microbial driven-weathering, directly by oxidoreduction reactions at the surface of minerals or indirectly by producing organic acids and chelating molecules (e.g., siderophores), are lacking [20, 21]. The same applied to the potential role of these communities in promoting soil aggregate stability through exopolysaccharide (EPS) and lipopolysaccharide (LPS) production [22, 23]. This emphasizes the importance of comprehensive studies to enhance our understanding of microbial functions in shaping arid and semiarid soils, particularly under changing climatic conditions.

Climate changes largely influence soil organic matter decomposition, mineral weathering, structure formation, and solute transport, thereby impacting the development and activity of soil microorganisms [24–26]. While climate change often highlights drought, studies also project extreme precipitation and humidification trends in arid regions like Northwest China and the Atacama Desert [27–29]. Differences in water availability have been shown to affect C-fixation rates along a precipitation gradient in grasslands, with the Calvin cycle prevailing in drier conditions and the rTCA predominant in humid ones [30]. Hydration in arid shrubland biocrusts and microcosm experiments with arid soils stimulates hydrogen (H_2_) oxidation, photosynthetic C-fixation, and, to a lesser extent, dark C-fixation [31, 32]. However, other studies show that the functional potential remains resilient and consistent, even with soil moisture variations [33, 34]. These investigations primarily examine the effects of moisture changes on microbial communities in arid environments [35] but sparsely address this impact on soil formation processes.

The Atacama Desert, renowned as one of Earth's driest regions, offers a natural laboratory to study the role of microorganisms in soil formation and stabilization. It hosts active in situ microbial communities in both endolithic and bare soils [4, 36, 37], which rapidly respond to episodic moisture increases, effectively utilizing available C and increasing mineralization activity [38]. Despite this response, a microcosm experiment simulating humid conditions revealed no discernible impact on soil formation by microorganisms alone within short time scales, which was attributed to experimental limitations that failed to capture small-scale microbial effects [39]. Thus, a direct assessment of microbial functionality is needed to understand its short-term role in soil formation. The advent of metagenomics and metatranscriptomics enables a comprehensive exploration of microbial genetic diversity and functions, which is crucial for understanding soil development. Functional metatranscriptomic analyses in the benthic coastline and halite nodules have primarily focused on pathways associated with cell maintenance, basic metabolic activities, and oxygenic photosynthesis [40, 41]. No studies, though, have directly explored microbial roles in soil formation in the Atacama Desert through metatranscriptomics despite the crucial functions of soils as nutrient reservoirs and biosphere habitats.

The present study aimed to investigate the microbial taxa actively transforming soils from arid and semiarid ecosystems under climate change towards humid conditions. Additionally, we characterize key microbial processes during soil transformation from arid to humid and how the associated gene expression differs between arid and semiarid sites under simulated humid climate conditions. Samples were collected from the arid site of Pan de Azúcar in the Atacama Desert and Santa Gracia, a semiarid zone adjacent to it, allowing us to study microbial interactions and initial soil-forming processes under different moisture and salinity conditions while sharing similar granitoid rock [24]. We also compared bare soils with soils containing plants, simulating more advanced stages of soil formation [42]. We hypothesize that a humid climate enhances gene transcription related to soil formation. We incubated soil samples under simulated humid climate conditions to test this hypothesis. We evaluated microbial responses at two and sixteen weeks to capture early and cumulative soil moisture effects. Continuing the work of Rodriguez et al. [39], we specifically analyzed metagenomic and metatranscriptomic data of soils, focusing on functional responses related to nutrient dynamics (C, N, P, and S), weathering, and soil aggregation.

## Methods

### Study sites and manipulation experiment

**Fig. 1:**
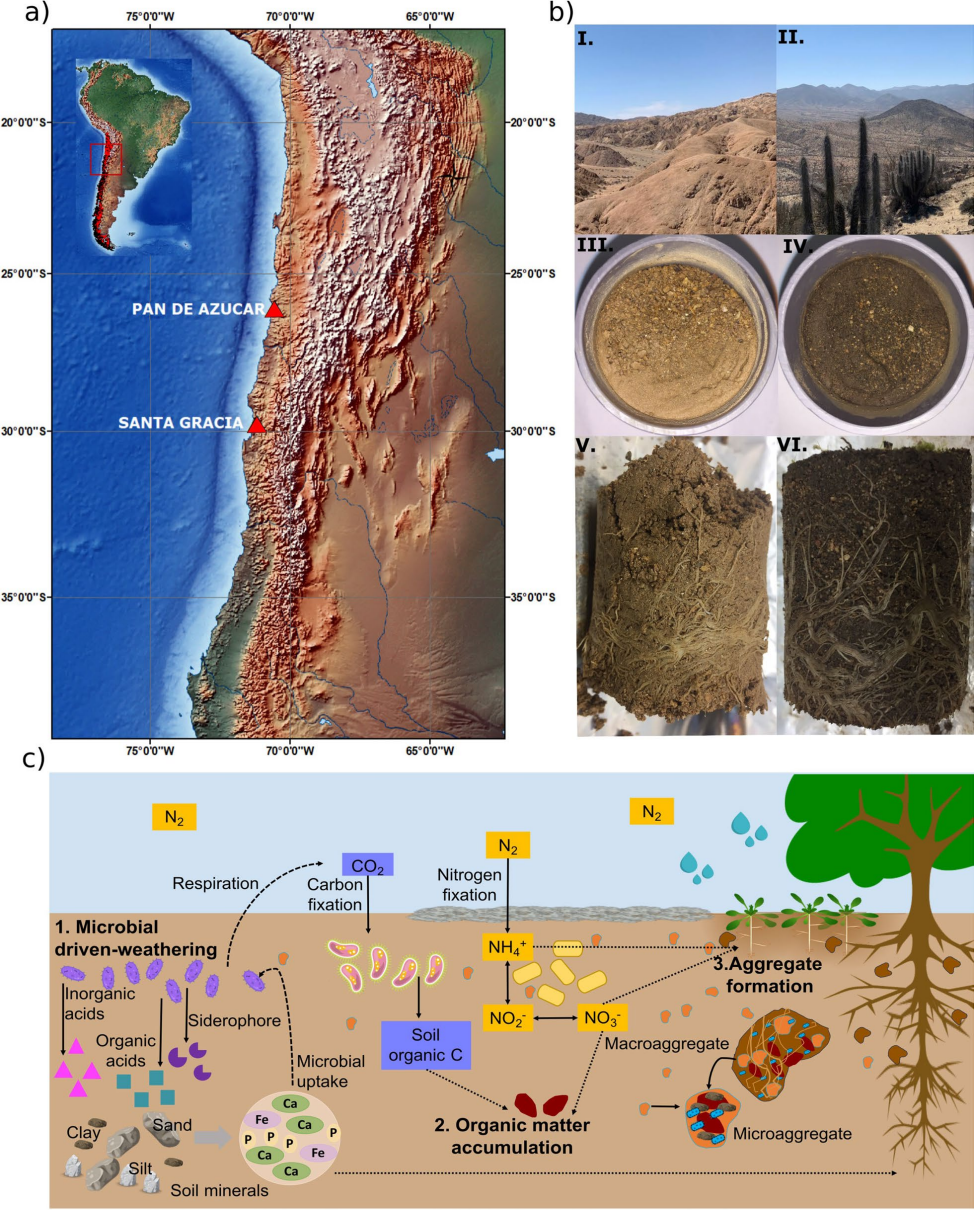
Key aspects of the study including **a** the location of study sites in the Chilean Coastal Cordillera, **b** the locations of Pan de Azúcar and Santa Gracia (**I** and **II**), along with microcosms in situ (**III** and **IV**) and with roots (**V** and **VI**) under simulated humid climate conditions at 16 weeks, and **c** the processes involved in soil formation, encompassing weathering, organic matter accumulation, and aggregation. In detail, **c** illustrates the rock and mineral weathering (1), C and N-fixation (2), and soil aggregate formation (3), all driven by microorganisms

The study area comprised two sites in the Chilean Coastal Cordillera (Fig. 1a), corresponding to Pan de Azúcar National Park (PA; 26° 06′ 35″ S, 70° 32′ 57″ W) and Santa Gracia Natural Reserve (SG; 29° 45′ 15″ S, 71° 10′ 3″ W). The geological composition at both study sites consists of granitoid rocks, ensuring a uniform parent material for comparative analysis [24, 43]. The climate conditions in PA are characterized by an arid climate with minimal precipitation and primarily endorheic water sources, where the mean annual precipitation and mean annual temperature are 12 mm and 16.8 °C, respectively [44]. In contrast, SG is in a semiarid climate and experiences coastal fog and winter precipitation. The mean annual precipitation is 66 mm, and the mean annual temperature is 13.7 °C [44]. These climate classifications are based on Bernhard et al. [24], which characterized each site.

The sampling campaign, conducted in March 2019 (end of the Austral summer), involved collecting soil samples from five vegetation-free plots at each site. These samples were sieved to 2 mm, homogenized, and stored at 4 °C before the experiment. A microcosm experiment was conducted to stimulate the transition from initial arid soils to developed soil ecosystems under humid conditions. Briefly, the experiment simulated the daily temperature of January (18.3/11.2 °C Day/night), photoperiod (14/10 h day/night), and moisture fluctuations (approximately 1.469 mm per year) of Nahuelbuta National Park (37° 48′ 25″ S, 73° 00′ 46″ W). This area was selected for its location in the Coastal Cordillera, known for well-developed deep soils with a stable soil structure [24]. Further details of the experimental setup and design can be found in Rodriguez et al. [39].

In the present study, we focused on soil samples from the A horizon, capturing the most biologically active layer at depths of 0–2 cm for PA and 0–3 cm for SG, reflecting the thickness of the A horizon at each site. Soils of each site were subjected to two treatments (Fig. 1b): (1) in situ from bare soil and (2) soil samples containing the pioneer plant *Helenium aromaticum* (Hook.) L.H. Bailey to simulate later stages of soil development. Samples from each treatment were assessed at three time points: the start of the experiment (Time 0), after two weeks (Time 1), and after sixteen weeks (Time 3) of simulated humid climate conditions. The number of biological replicates varied at each time point, with two for Time 0 and three for Time 1 and 3. Based on our previous 16S rRNA amplicon sequencing results [39], which showed low variability between in situ and plant treatments (Additional file 1: Fig. S1), we focused exclusively on the in situ treatment for metagenomic sequencing. Conversely, we included both treatments for metatranscriptome analysis to capture potentially higher variability. In total, we obtained sixteen samples for DNA analysis from two sites (PA and SG) and one treatment (in situ), collected at three time points (Time 0, Time 1, and Time 3) and stored at − 20 °C. Additionally, twenty-four soil samples were collected, representing two sites (PA and SG), two treatments (in situ and plant), and two sampling times (Time 1 and 3), each with three biological replicates for RNA analysis. These samples were promptly placed into falcon tubes with liquid nitrogen and stored at − 80 °C for subsequent use. Time 0 samples were excluded from RNA analyses because they were previously stored at − 20 °C.

### Soil physicochemical analysis

The detailed methodology and results of physicochemical analyses are presented in Rodriguez et al. [39]. From the parameters discussed in their publication, this study focuses on thirteen specific parameters for further investigation (Additional file 2: Table S1). These parameters encompass pH, electrical conductivity (EC), total sulfate, phosphate, sodium, and nitrate, total organic carbon (TOC), total nitrogen in the bulk soil (N_bulk_), aggregate size distribution (macroaggregates and large and small microaggregates), total carbon (C_t_) and total nitrogen (N_t_) categorized by aggregate size, and the mean weight diameter (MWD). In summary, soil pH and EC were measured using a WTW pH 340 with a Sentix 81 electrode and a conductivity meter (LE703, Mettler Toledo, United States). Inorganic ions were analyzed via ion chromatography. TOC and N_bulk_ were measured using a Flash HT 2000 Organic elemental analyzer. Aggregate size distribution was determined using sieve analysis in a modified Casagrande apparatus (Mennerich Geotechnik, Hannover, Germany), and C_t_ and N_t_ were measured based on size distribution using a CHNSO elemental analyzer (HEKAtech Euro EA, Wegberg, Germany).

### Enzymatic activity assays

We conducted an enzymatic activity hydrolysis assessment using fluorescein diacetate (FDA), following the protocol described in Schulze-Makuch et al. [4] with minor modifications. In summary, we mixed 2 g of soil with 25 mL of sterile potassium phosphate buffer (60 mM, pH 7.6) and added 0.25 mL of FDA solution (4.9 mM FDA dissolved in acetone). The mixture was then incubated at 37 °C for 3 h, and we stopped the enzyme reactions by adding 1 mL of acetone. Next, we centrifuged the sediment slurry (8800 g, 5 min), filtered the supernatant through a PP syringe into a new tube, and measured the fluorescence intensity at 490 nm using a spectrophotometer (HACH DR 39000, USA). We determined endpoint concentrations using a standard curve constructed with sodium fluorescein (Additional file 1: Fig. S2).

### DNA/RNA extraction, quality control and sequencing

The DNA extraction for PA samples involved 0.5 g of soil, while SG samples used 0.25 g, following the PowerSoil DNA isolation kit protocol (Qiagen, Hilden, Germany). Before extraction, biological replicates were homogenized to capture all variation from the replicates into a single consolidated sample (Additional file 1: Fig. S3). Each sample was then extracted in duplicate and mixed at the end of the extraction process. Consequently, six samples were generated for DNA sequencing, covering two sites (PA and SG), one treatment (in situ), and three sampling times (Time 0, 1, and 3). Similarly, for RNA extraction, biological (3 samples) and technical replicates (2 samples) were homogenized before and after the extraction, respectively, leading to a total of eight samples representing two sites (PA and SG), two treatments (in situ and plant), and two evaluation times (Time 1 and 3). Total RNA extraction was performed on 4 g of soil for PA and 2 g for SG in each sample, using the soil RNeasy PowerSoil Total RNA kit (Qiagen, Hilden, Germany). Finally, following the manufacturer's protocol, we employed the TURBO DNA-free Kit (Invitrogen, Thermo Fisher, Berlin, Germany) to eliminate any residual DNA contamination from RNA samples before further quantification and library preparation.

The quality and quantity of DNA and RNA were assessed fluorometrically using Genomic DNA ScreenTapes and High-sensitive RNA ScreenTapes on the Agilent 4150 TapeStation system (Agilent Technologies, USA) and Qubit 2.0 fluorometer (Thermo Fisher Scientific, United States). For metagenomic and metatranscriptomic analyses, Eurofins Genomic Germany GmbH (Ebersberg, Germany) conducted library preparation and RNA/DNA sequencing on an Illumina NovaSeq6000 platform, targeting 30 million paired-end reads for DNA and 40 million for RNA with a length of 150 nt.

### Data processing

#### Metagenome analysis

The metagenome data was processed using the ATLAS pipeline v2.12.0 [45], which includes an extended workflow for quality control, contig assembly, gene prediction, functional annotation, binning of contigs into MAGs, and taxonomic annotation of the MAGs. Among the included tools are several standard tools, e.g., metaSPAdes v3.15.3 for read assembly [46], eggNOG mapper v2.1 and eggNOG database v5.0 for functional gene annotation [47], maxBin2 v2.2 [48], metabat2 v2.15 [49] and DAS_Tool v1.1.4 [50] for binning and refinement of MAGs, checkM v1.1.10 for MAG quality assessment [51], and GTDBtk tool version 2.1.1 with GTDB database release207 for taxonomic annotation of the MAGs [52]. Default parameters were used, except for RAM (up to 1.5 TB), CPU/threads (up to 80 threads), and the minimum contig length, which was set to 500 bp. To increase the sensitivity of the assembly of low-abundant species shared between samples, we constructed a merged sample from the six metagenome samples. This co-assembly-like sample was processed together with the other samples.

Gene taxonomy assignment to specific phyla was done using the phylum of the closest hit given by eggNOG results from above.

#### Metatranscriptome analysis

Metatranscriptomes were quality-controlled using the ATLAS pipeline, and high-quality reads were mapped to all detected genes from the metagenomes using Bowtie2 v2.3.4.1 [53]. The functional annotation with eggNOG also annotates KEGG orthology IDs (KOs). Additional file 2: Table S2 includes all the corresponding genes and KO numbers that were part of the analysis corresponding to enzymes involved in soil development (Fig. 1c). Differential gene expression analysis due to site (adjusted *p-value* < 0.05) over the simulation was performed using R 4.0 and the DESeq2 package v1.34 [54]. All details required for reproducibility are available in a public repository on GitHub (https://github.com/AlexanderBartholomaeus/SoilSim).

#### Taxonomic assessment of metagenome and metatranscriptome samples

The metatranscriptomes were not depleted for rRNA, which allows taxonomic assessment based on the rRNA. The quality-controlled reads (from ATLAS, see above) were mapped to the SILVA rRNA database v138 [55] using Bowtie2 v2.3.4.1 [53]. Taxonomic nomenclature might vary throughout the manuscript due to using the GTDB database for taxonomic annotation of the MAGs, the SILVA database for species abundance quantification using the rRNA reads, and gene annotation taxonomy classification by the eggNOG database and the NCBI taxonomy.

#### Gene abundance normalization

Gene counts for metagenomes and transcriptomes were normalized by calculating RPMs (reads per million mapped reads) using R v4.0.5, where the total gene counts for each sample are the mapped reads of all genes: RPM = (gene or transcript read count)/(total mapped reads of the sample) * (1,000,000 reads). A table with the total mapped read is added as Additional file 2: Table S3.

### Data analysis

We performed data analysis and plot generation using R v4.0.5 [56]. The vegan R package [57] conducted Principal Coordinates Analysis (PCoA) analyses, with inter-sample distances calculated using the Bray–Curtis dissimilarity metric. Statistical differences in composition were assessed via PERMANOVA, utilizing the 'adonis' function. Graphics were created using ggplot2 v3.4.4 [58].

## Results

### Soil physical and chemical properties

Soil parameters revealed distinct soil development between PA and SG (Additional file 2: Table S1), with significant differences (*p-value* < 0.05; Additional file 2: Table S4). PA demonstrated less developed soil attributes, as evidenced by higher pH (7.8 compared to 6.6) and sodium (44.9 compared to 4.1 mg L^−1^), along with lower TOC (0.14% compared to 0.93%), N_t_ (0.03% compared to 0.08%), aggregate stability (MWD of 0.67 compared to 0.75 mm) and plant development than SG. In contrast, SG exhibited progressive soil formation characterized by organic matter accumulation, decreased pH, and increased aggregate stability and vegetation cover. PA exhibited a more pronounced response in physicochemical parameters under simulated climate change than SG over time. PA showed decreases in pH (from 7.9 to 7.7), EC (from 1809 to 65.5 μS cm^−1^), sodium (from 108.8 to 9.1 mg L^−1^), nitrate (from 12.3 to 1.2 mg L^−1^), and sulfate (from 46.6 to 4.7 mg L^−1^) concentrations from T0 to T3. For SG, there was a decrease in EC (from 121.9 to 26.3 μS cm^−1^), with decreases in TOC (1.1 to 0.9%). Despite no significance, both sites experienced macroaggregate increases and large-microaggregate decreases, contributing to a higher mean weight diameter (MWD) from T0 to T3, indicative of enhanced soil aggregate stability. Moreover, microorganism-plant treatments resulted in a prominent rise in macroaggregates and MWD compared to in situ treatments, with MWD values of PA (0.78 vs. 0.62 mm) and SG (0.87 vs. 0.76 mm).

### Sequencing data insights

We generated six metagenomic and eight metatranscriptomic datasets to characterize the community structure, the functional potential encoded, and transcriptomic activity. The assembled metagenomes resulted in 5,159,371 contigs with 5,064,696,610 total bases (Additional file 2: Table S5). Thirty-four high-quality MAGs (completeness >90% and contamination <5%) were extracted after binning and refinement (Additional file 2: Table S6).

**Fig. 2:**
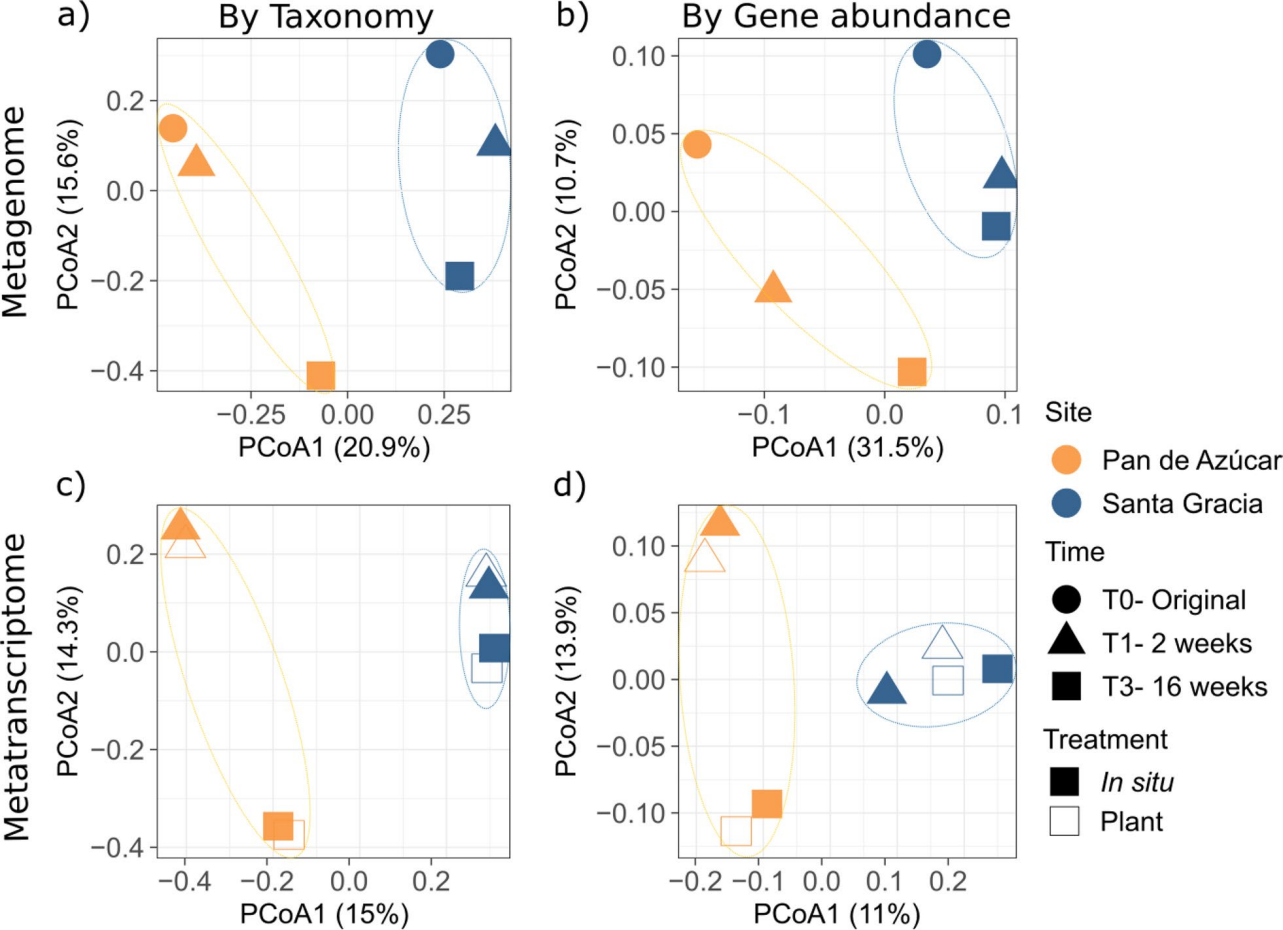
PCoA using Bray–Curtis distance on the metagenome and metatranscriptome. **a** PCoA based on the taxonomic abundance in the metagenome inferred from read mapping the SILVA database, **b** PCoA based on gene abundance in the metagenome inferred from read counts per gene, **c** PCoA based on the taxonomic abundance in the metatranscriptome inferred from read mapping the SILVA database, and **d** PCoA based on gene abundance in the metatranscriptome inferred from read counts per gene. Samples are categorized by site (Pan de Azúcar and Santa Gracia) and treatment (in situ and plant) at the initial time, after two and 16 weeks

We compared the microbial community structure and functions between the two sites by analyzing the taxonomic abundance of species (inferred by mapping the reads to the SILVA database) and the transcriptional profiles of all genes in the metagenomes and metatranscriptomes with PCoA based on the Bray–Curtis dissimilarity matrix (Fig. 2). We observed no significant variations in microbial taxa among different sites (n = 3). However, transcriptional profiles revealed significant differences in gene expression between sites (PA and SG), as determined by PERMANOVA (n = 4, *p-value* 0.03). Due to the limited sample size, statistical significance could not be assessed from T1 to T3. Still, the PCoA analysis revealed more pronounced temporal shifts in the taxonomic abundance of species and transcriptional profiles in PA compared to SG. Regarding treatments, PA exhibited less variation when comparing different treatments at the same sampling time. In contrast, we observed slight differences among treatments in SG, where plant treatments showed higher similarity than in situ treatments when comparing T1 and T3.

### Taxonomic structure of the microbial communities

**Fig. 3:**
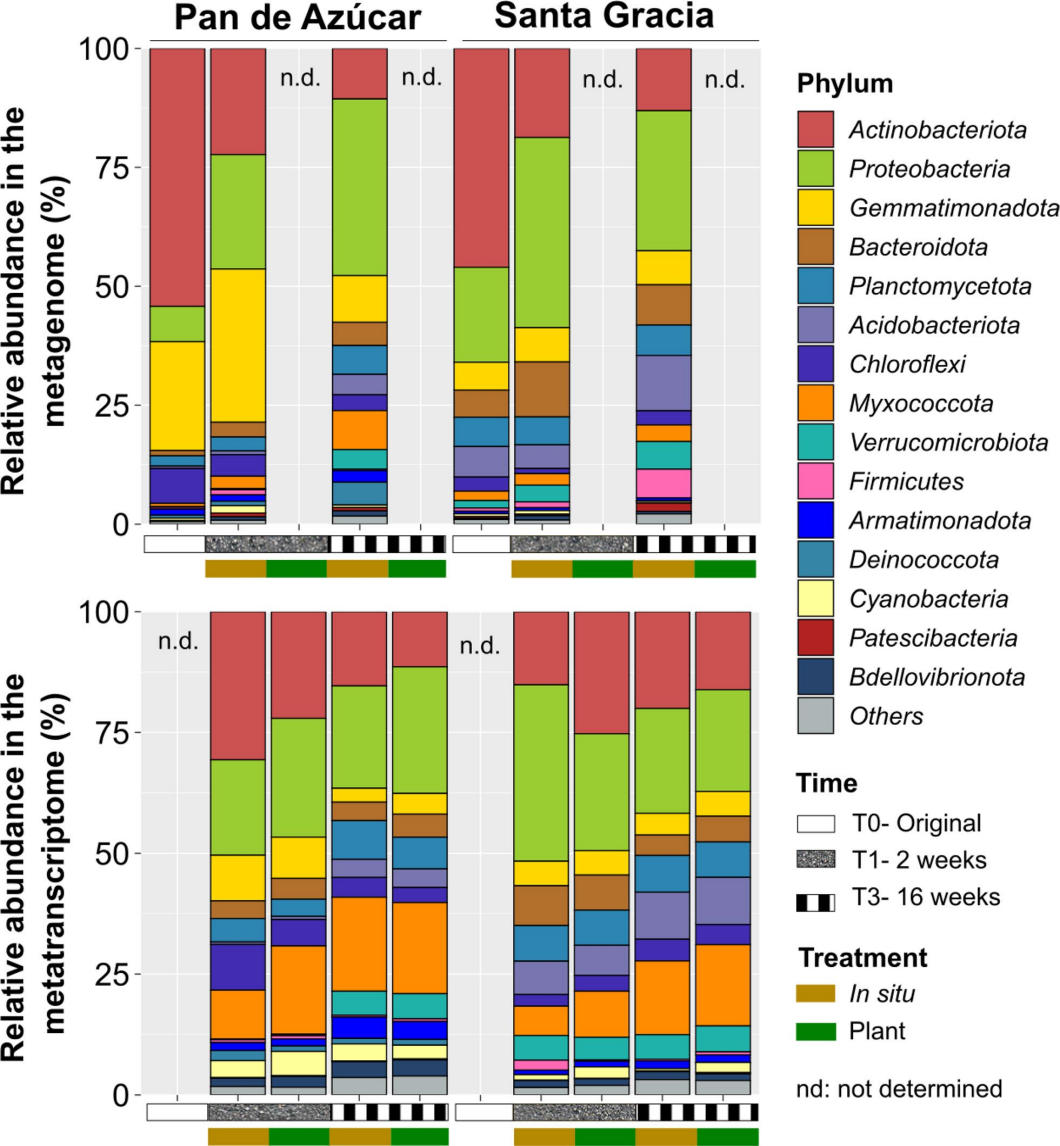
Proportion of the most abundant phylum-level taxa determined by **a** metagenomic and **b** metatranscriptomic analysis. Samples are categorized by site (Pan de Azúcar and Santa Gracia) and treatment (in situ and plant) at the initial time, after two and 16 weeks. n.d. samples mean not determined

The taxonomic composition was predicted using both metagenomes and the taxonomic profile of the transcriptionally active community using the Silva database (Fig. 3, Additional file 2: Table S7). Subsequent analyses were based on relative abundances. The taxonomic composition based on metagenomic data revealed that bacterial communities dominated the soil microbiota, constituting an average relative abundance of 99.7%, while archaea constituted a much smaller proportion, with an average relative abundance of 0.3%. The dominant phyla across all samples were *Actinobacteriota*, *Proteobacteria*, *Gemmatimonadota*, *Bacteroidota*, and *Planctomycetota*, collectively representing 78.7% of all taxa sequences. In the bacterial domain of PA, *Actinobacteriota* (29.1%), *Proteobacteria* (22.9%), and *Gemmatimonadota* (21.6%) dominated. For SG, the dominant phyla were *Proteobacteria* (29.8%), *Actinobacteriota* (25.9%), and *Bacteroidota* (8.6%). Both sites showed an increase in *Proteobacteria* (from 7.4 to 37.1% in PA and from 20 to 29.5% in SG) and a decrease in *Actinobacteriota* (from 54.2 to 10.6% in PA and from 46 to 13.1% in SG) from T1 to T3. The most abundant families in PA included *Longimicrobiaceae* (18.7%), *Sphingomonadaceae* (10.7%), and *67–14* (7.2%). Notably, *Sphingomonadaceae* increased while the others decreased over time. In contrast, SG showcased families such as *Geodermatophilaceae* (7.2%), *Sphingomonadaceae* (6.6%), and *Gemmatimonadaceae* (5.3%) as the most abundant ones. *Geodermatophilaceae* decreased, and the rest remained stable from T0 to T3. Regarding archaea, SG exhibited a higher representation (0.4%) than PA (0.1%). *Crenarchaeota* dominated the archaea domain in both PA (0.1%) and SG (0.4%), primarily represented by the family *Nitrososphaeraceae* (0.09% in PA and 0.4% in SG). While *Nitrososphaeraceae* showed a slight increase in PA, it showed a slight decrease in SG over time.

The transcriptionally active community provided valuable insights into active members based on gene expression data. Similar to the metagenomes, bacteria constituted the predominant group (99.3%), while archaea accounted for a much smaller proportion (0.7%). The dominant active phyla across all samples were *Actinobacteriota*, *Proteobacteria*, *Myxococcota*, *Planctomycetota*, and *Bacteroidota*, collectively representing 70.4% of all taxa sequences. In the PA soil, the most active phyla were *Proteobacteria* (22.9%), *Actinobacteriota* (19.9%), *Myxococcota* (16.7%), and *Gemmatimonadota* (6.3%). Conversely, in SG, the active phyla included *Proteobacteria* (25.9%), *Actinobacteriota* (19.1%), *Myxococcota* (11.9%), and *Acidobacteriota* (7.7%). Notably, *Myxococcota* was transcriptionally abundant in both sites despite not being taxonomically abundant, and its abundance increased over time. Moreover, *Proteobacteria* remained stable (from 22.2 to 23.7%) as the other phyla decreased in PA over time, whereas in SG, *Proteobacteria* decreased (from 30.3 to 21.4%) while the others stayed steady. At the family level in PA, notable families were *Myxococcaceae* (6.7%), *Haliangiaceae* (4%), and *Longimicrobiaceae* (5.3%). Among them, only *Haliangiaceae* increased from T1 to T3, while the others showed a decrease. In SG, active families included *Geodermatophilaceae* (4.2%), *Comamonadaceae* (3.7%), and *Oxalobacteraceae* (3.6%). From them, *Oxalobacteraceae* decreased, and the rest remained stable over time. SG exhibited a more active archaeal community (0.8%) than PA (0.6%), with *Crenarchaeota* dominating in both soils (0.7%), primarily represented by the family *Nitrososphaeraceae* (0.6% in PA and 0.8% in SG), showing no changes over time for both sites.

### Functional characterization of microbial community response

**Fig. 4:**
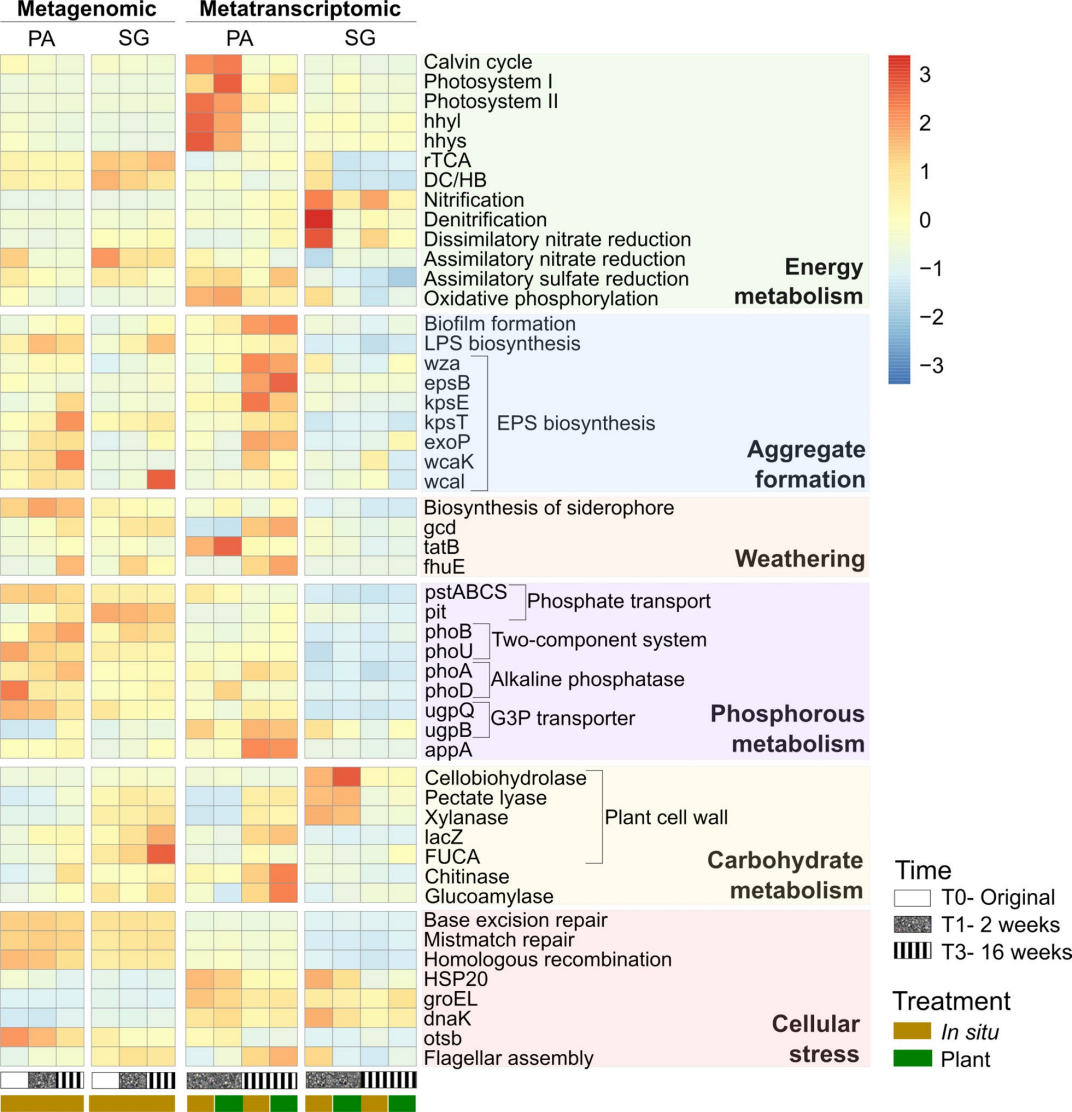
Heatmap representing the gene expression levels of pathway, module, or key genes associated with soil formation over 16 weeks. The heatmap is generated from selected KEGG orthologs using normalized counts from metagenomic and metatranscriptomic data. Samples are categorized by molecular analysis (metagenome and metatranscriptome), site (Pan de Azúcar-PA and Santa Gracia-SG), and treatment (in situ and plant) at the initial time, after two and 16 weeks. The color gradient represents the relative abundance of each gene, module, or pathway in the metagenomes and the corresponding transcripts in the metatranscriptome, ranging from low (blue) to high (red) levels. A comprehensive list of the key genes related to each pathway and module can be found in Additional file 2: Table S2

**Fig. 5:**
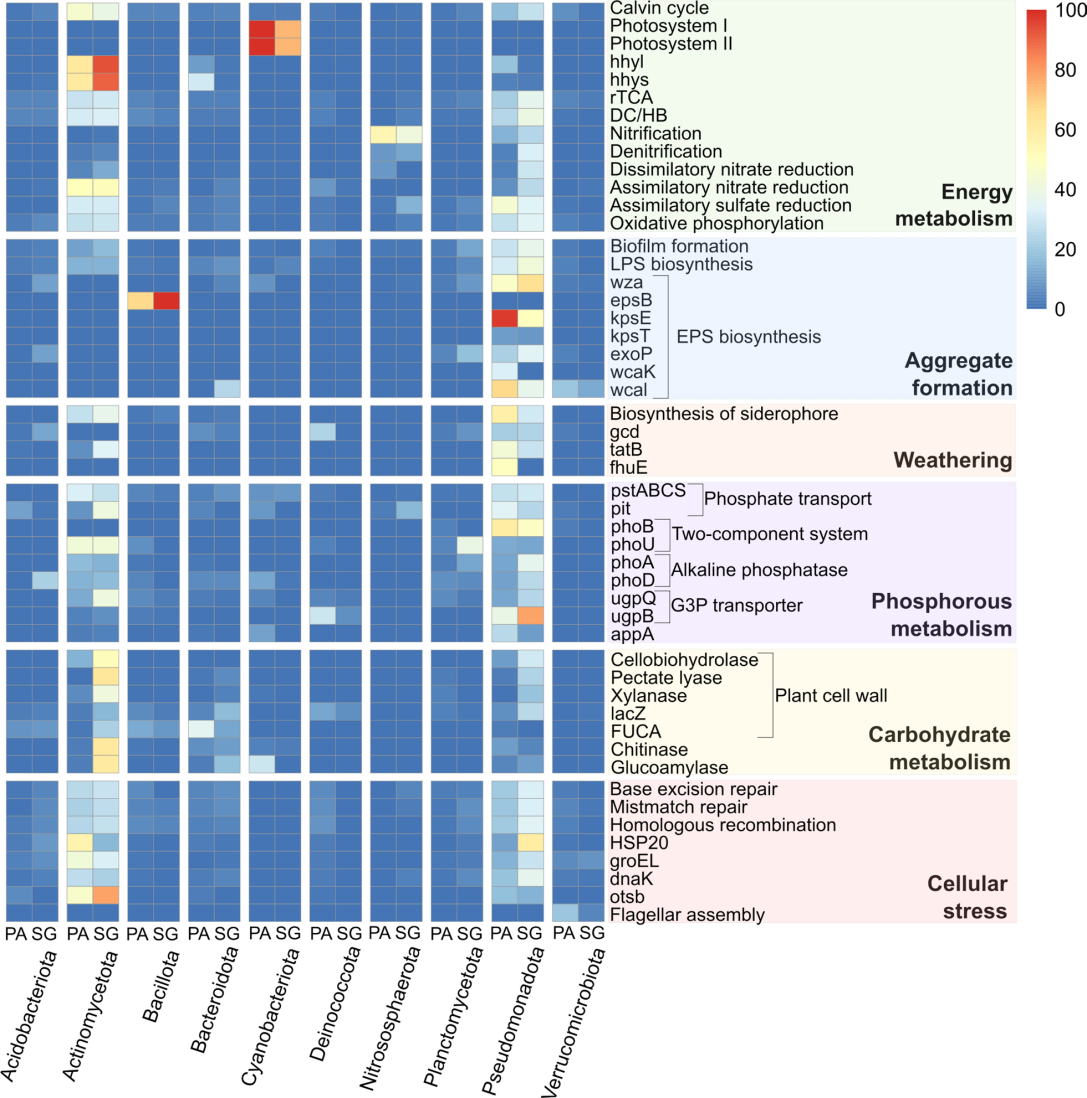
Heatmap of phyla abundance and its association with soil formation functions in the metatranscriptome. Comparison of Pan de Azúcar (PA) and Santa Gracia (SG) samples, evaluating the top 10 phyla abundance for each site and function. The color gradient represents the relative contribution of each phylum to transcripts associated with different genes, modules, or pathways, ranging from 0% (blue) to 100% (red)

Our transcriptional results demonstrate the activity of at least a fraction of the microbial community, with mRNA representing an average of 9.7% of the sequencing data (Fig. 4, Additional file 2: Tables S2, S3). The soil samples exhibited enrichment in main functional processes related to carbohydrate metabolism and genetic information processing (DNA replication). These processes were significantly more abundant in SG than in PA. In contrast, PA displayed a higher abundance in genes associated with homologous recombination, base excision repair, and mismatch repair traits. Moreover, specific stress resistance and damage repair genes exhibited high transcription in both sites. Specifically, the chaperon coding genes *HSP20*, *groEL* (HSP60), *dnaK* (HSP70), and the trehalose synthesis gene *otsB* were initially abundant but showed a slight decrease over time in PA. In SG, only *HSP20* and *dnaK* decreased. The phyla assigned to these pathways and genes are *Actinomycetota* for *otsB*, or *Pseudomonadota* and *Actinomycetota* for homologous recombination, base excision repair, mismatch repair traits, *dnak*, *groEL*, and *HSP20* (Fig. 5). In addition, an increase in the expression of dispersion genes such as flagella and gliding motility (pilus assembly) was observed in PA from T1 to T3, while SG showed no trend in expression changes over time.

#### Microbial-driven weathering

We examined the gene expression patterns for P-solubilization and iron (Fe) uptake (Figs. 4, 5). The gene *gcd*, encoding quinoprotein glucose dehydrogenase responsible for solubilizing inorganic P, was identified in all samples, with higher relative abundances in PA than SG. The relative abundance of this gene, found in *Pseudomonadota*, *Deinococcota*, and *Bacteroidota*, remained stable in SG (mean 0.03) but increased from T1 to T3 in PA (from 0.007 to 0.09). On the other hand, siderophore biosynthesis, crucial for Fe uptake from the environment into cells, was significantly more highly transcribed in PA than in SG (*p-value* < 0.05). Although siderophore biosynthesis did not change over time, plant treatments showed higher transcription than in situ samples in T1 and T3. Furthermore, the *fhuE* gene, involved in bacterial Fe uptake via specialized outer membrane proteins, exhibited increased expression from T1 to T3 in PA. Both the gene for siderophore biosynthesis and the *fhuE* gene were mainly found in *Pseudomonadota*.

#### Exo- and lipopolysaccharide metabolism

We also evaluated the gene expression involved in EPS and LPS biosynthesis and transport, which are essential for bacterial biofilms and soil aggregate formation (Figs. 4, 5). The predominant genes involved in EPS polymerization and export were *wza*, *wzc*, and *exoP*. Both *wza* and *exoP* increased over time in PA from T1 to T3 but remained stable in SG, primarily contributed by *Pseudomonadota* (mainly *Rhodospirillales* and *Sphingomonadales*), *Acidobacteriota*, and *Planctomycetota*. In contrast, *wzc* remained constant at both sites and was primarily contributed by *Pseudomonadota*. We also analyzed genes involved in the capsular polysaccharide export, specifically *KpsE* and *KpsT*, which increased significantly from T1 to T3 in PA and were more abundant in PA than in SG. These genes were exclusively assigned to *Pseudomonadota*, particularly *Sphingomonadales* and *Vibrionales*, and correlated positively with small microaggregates (Additional file 1: Fig. S4). For the EPS colanic acid, the *wcaJ*, corresponding to the initiator of colanic acid biosynthesis, and *wcaK*, which is involved in colanic acid/amylovoran, were also identified. While the abundance of *wcaJ* remains stable over time in both sites, *wcak* showed an increase from T1 to T3 in PA. *Pseudomonadota* predominantly contributes to *wcaK*, whereas *wcaJ* is additionally associated with *Bacteroidota*.

Genes involved in LPS metabolism exhibited stable transcription over time at both sites (Additional file 2: Tables S2, S8). The *lptA* gene associated with LPS biogenesis was primarily found in *Pseudomonadota* (*Sphingomonadales* and *Xanthomonadales*) and *Acidobacteriota*. The *wzt* gene, encoding an ATP-binding protein for LPS O-antigen transport, was significantly more abundant in PA than SG, carried by *Pseudomonadota* (*Chromatiales* and *Xanthomonadales*), *Acidobacteriota* and *Cyanobacteriota*. Furthermore, genes *lptBFGC*, encoding components of the LPS export permease, were present in *Gemmatimonadota* and *Pseudomonadota* (*Xanthomonadales*, *Sphingomonadales*, *Acidithiobacillales*, and *Caulobacterales*).

#### Carbon metabolism

Prominent C-fixation pathways included the rTCA (Arnon-Buchanan cycle), dicarboxylate-hydroxybutyrate cycle (DC/HB), 3-hydroxypropionate bicycle (3HP), and Calvin cycle (Figs. 4, 5, Additional file 2: Tables S2, S8). The rTCA cycle was the most abundant, fixing two CO_2_ molecules to synthesize a single acetyl CoA molecule. From the genes involved in this cycle, carboxylases alpha-ketoglutarate oxidoreductase (*KorA* and *KorB*), isocitrate dehydrogenase (*idh1*), phosphoenolpyruvate carboxylase (*ppc*), and pyruvate: ferredoxin oxidoreductase (*porA* and *porB*) exhibited active transcription. However, genes encoding citryl-CoA synthetase (*ccsA* and *ccsB*), citryl-CoA lyase (*ccl*), and ATP citrate-lyase (*aclA* and *aclB*), responsible for the conversion of citrate to oxaloacetate, were not identified in the metatranscriptomes. Nevertheless, oxaloacetate may be formed via pyruvate, as the genes encoding enzymes involved in this conversion are present. While the pathway exhibited only a slight increase in PA from T1 to T3, there was a remarkable upregulation in the transcription of *korA* and *korB* over time. In contrast, SG showed stability in the transcription levels of these genes. *Pseudomonadota* significantly contributed to the transcription of all cycle genes, including orders such as *Xanthomonadales*, *Rhodospirillales*, and *Sphingomonadales*. Moreover, also *Planctomycetota* (*korB*), *Actinomycetota* (*korA*, *korB*, *ppc*, and *idh1*), *Nitrospirota* (*porA* and *porB*), and *Bacillota* (*porB* and *idh1*) contributed to specific genes.

The Calvin cycle, the most widespread pathway for CO_2_ fixation, exhibited complete gene presence and high utilization in PA compared to SG. In PA, key genes such as phosphoribulokinase (*prk*) and the large (*rbcL*) and small chains (*rbcS*) of Ribulose-1,5-bisphosphate carboxylase (RuBisCO) were significantly more abundant (*p-value* < 0.05) and primarily assigned to *Actinomycetota*, with lesser contributions from *Pseudomonadota* and *Cyanobacteriota*. These genes correlated positively with microaggregates and negatively with TOC and N_t_ in microaggregates (Additional file 1: Fig. S4). While the Calvin cycle genes involved remained stable in SG, a notable decrease was observed in PA from T1 to T3, reaching lower levels than rTCA. In addition, we evaluated H_2_ oxidation genes that can support the Calvin cycle, showing a decrease from T1 to T3 in the large (*hhyl*) and small (*hhys*) subunit hydrogenase genes mainly carried by *Actinomycetota*.

The Reductive Acetyl-CoA pathway (Wood-Ljungdahl cycle) was incomplete in both sites due to the absence of anaerobic carbon-monoxide dehydrogenase and the acetyl-CoA synthase. Similarly, we identified the key enzyme in the 3HP pathway, acetyl-CoA/propionyl-CoA carboxylase (encoded by *accABC* and *ppcB* genes), but malonyl-CoA reductase was absent. In the hydroxypropionate-hydroxybutyrate cycle (HP/HB), propionyl-CoA carboxylase (similar or identical to acetyl-CoA carboxylase) showed stability in SG over time and a slight decrease in PA, primarily carried by *Pseudomonadota*, *Actinomycetota*, and *Gemmatimonadota*. Moreover, *accC* correlated positively with small microaggregates and negatively with TOC and N_t_ in small microaggregates (Additional file 1: Fig. S4). Regarding DC/HB, the critical enzyme 4-hydroxybutyryl-CoA dehydratase (*abfD*) was actively transcribed in both sites, predominantly by *Nitrososphaerota* and *Pseudomonadota*. Over time, the *abfD* transcription increased slightly in PA and decreased in SG. These results indicate that both HP/HB and DC/HB for CO_2_ fixation may occur, given that the enzymes that catalyze the two main metabolic reactions are active.

Unfortunately, our study could not identify the key enzyme for archaeal methane metabolism, the methyl-coenzyme M reductase complex (*mcr*). Although the particulate methane monooxygenase (*pmoA*) gene was detected in all samples, indicating methane oxidation, crucial genes for the soluble methane monooxygenase (*mmoX*) and the methanol dehydrogenase (*mxaF*) subunits were not present.

#### Carbohydrate metabolism

We identified genes for heterotrophic carbohydrate metabolism from diverse sources in soil samples (Figs. 4, 5, Additional file 2: Table S2). Cellobiohydrolase (*CBH2*), which degrades plant cellulose, showed higher transcription in SG than PA and decreased throughout the experiment in both soils and correlated positively with TOC, N_bulk_, and N_t_ in all soil aggregates (Additional file 1: Fig. S4). This gene was predominantly linked with *Actinomycetota* and *Pseudomonadota* in SG, with less taxonomic identification in PA. Enzymes for hemicellulose degradation, including beta-galactosidase (*lacZ*), alpha-N-arabinofuranosidase (*abfA*), and alpha-l-fucosidase (*FUCA*), displayed diverse trends. *lacZ* and *FUCA* increased from T1 to T3 in PA, with no clear trends in SG. *Bacteroidota*, *Bacillota*, *Pseudomonadota*, and *Actinomycetota* contributed to these enzymes. In contrast, *abfA* showed higher abundance in SG, particularly in plant treatments, and is associated with *Actinomycetota*, *Bacillota*, and *Bacteroidota*. Moreover, pectin (pectate lyase) and xylan (xylanase) degradation, mainly conducted by *Actinomycetota*, increased in PA from T1 to T3, while transcription decreased during the same period in SG. The chitin degradation by the chitinase enzyme increased in PA but decreased in SG, to which *Actinomycetota* and *Bacteroidota* contributed. Finally, the starch-degrading enzyme glucoamylase, mainly carried by *Cyanobacteria* (PA) and *Bacteroidota* (SG), increases over time in PA while stable in SG.

#### Nitrogen fixation and metabolism

In the N metabolism pathway, denitrification, nitrification, and dissimilatory nitrate reduction (DNR) were predominant (Figs. 4, 5, Additional file 2: Tables S2, S8). In contrast, assimilatory nitrate reduction (ANR) was less abundant, and anaerobic ammonium oxidation (anammox) genes were not identified. Additionally, the N-fixation pathway (*nif* genes) was scarcely present in the metagenomes, showing a slight increase from T0 to T3 but with no activity in the metatranscriptome. These genes were predominantly in *Pseudomonadota* (e.g., order *Rhodospirillales*) and *Bacillota*.

We identified ANR genes in both PA and SG metagenomes. *nasA* catalyzes the conversion of nitrate (NO_3_^−^) to nitrite (NO_2_^−^), followed by *nirA* facilitating the conversion of NO_2_^−^ to ammonium (NH_4_^+^), with consistent transcription observed across all soil samples. However, *nasB* and *narB* genes exhibited lower representation in the metatranscriptome, with *narB* absent in PA. *Proteobacteria*, *Actinobacteriota*, *Euryarchaeota* were predominant in *nasA, nirA* and *nasB* gene expression. *Bacteroidota* and *Euryarchaeota* contributed to *narB* expression, showing no evident temporal changes. The NH_4_^+^ produced from ANR is further assimilated through glutamine synthetase (*glnA*), converting glutamate to glutamine. *Actinomycetota* and *Pseudomonadota* were the primary carriers of *glnA*, with stable expression across all samples.

The DNR pathway includes *nirB*, *nirD*, *nrfA*, and *nrfH* genes, which are present in all the metagenomes. *nirB*, converting NO_2_^−^ to NH_4_^+^, was significantly more abundant in SG than in PA, alongside active transcription of *nirD* in all samples. *nrfH* and *nrfA* were exclusively active in SG, increasing over time. *Pseudomonadota* dominated *nirB* gene expression in both sites, with *Actinomycetota* contributing in SG. For *nirD*, *Actinomycetota* and *Bacteroidota* were major contributors in SG, while *Nitrospirota* and *Chloroflexota* were in PA. *Verrucomicrobiota* exclusively contributed to *nrfA* in SG.

Denitrification genes (*narGHI*, *napAB*, *nirKS*, *norBC*, and *nosZ*) catalyze NO, N_2_O, and N_2_ gas production from NO_3_^−^ under anoxic conditions. All genes were actively transcribed in SG except *napB* and *narI* (only in SG T1 in situ), while *norB*, *nirS*, *napB*, and *narI* were not transcribed in PA. Notably, *napAB* and *narGHI* showed higher transcription in SG T1 in situ, decreasing to T3. *Pseudomonadota*, including the order *Rhodospirillales*, played a pivotal role in this pathway in SG, while *Nitrososphaerota* was crucial in *nirK* and *Actinomycetota* in *napA*.

The nitrification pathway involves ammonia oxidation and nitrite oxidation. Genes for ammonia oxidation, catalyzed by ammonia monooxygenase (*Amo*), which produces hydroxylamine, and its subsequent oxidation by hydroxylamine oxidoreductase (*Hao*) to NO_2_^−^, were identified in all the metagenomes. *amoABC* showed significantly higher abundance in the metatranscriptome of SG, with *Hao* absent in SG T3. SG T1 in situ samples displayed higher nitrification gene transcription, which diminished over time. In contrast, PA T3 showed a slight increase in the transcription of these genes. *Pseudomonadota* dominated this pathway, with contributions of *Nitrososphaerota* in *amoA* and *amoB* and *Actinomycetota* in *amoC*. Nitrification gene transcription abundance correlated positively with TOC and N_bulk_ (Additional file 1: Fig. S4).

#### Phosphorous metabolism

Genes related to soil P turnover included enzymes for solubilization of inorganic and organic P, uptake systems, transport, and regulatory systems (Figs. 4, 5, Additional file 2: Tables S2, S8). In addition to the *gcd* gene (see 3.4.1), both soils exhibited alkaline phosphatase genes (*phoD* and *phoA*), which were significantly more abundant in PA than SG and associated with *Pseudomonadota*, *Actinomycetota*, and *Acidobacteriota* (particularly *phoD* in SG). While alkaline phosphatases remained stable in SG, *phoA* increased from T1 to T3 in PA.

Genes for inorganic phosphate transporters (*pstSCAB*) and low-affinity phosphate inorganic transporters (*pit*) were present in all samples, with *pstSCAB* significantly more abundant in PA than in SG. *Actinomycetota* and *Pseudomonadota* were primarily associated with *pst* genes. The *pit* gene was linked to *Pseudomonadota* in both sites, while *Actinomycetota* and *Nitrososphaerota* were important in SG. While *pstB* and *pstS* genes remained stable in SG, they decreased in PA, and the *pit* gene slightly increased from T1 to T3. Moreover, genes for a two-component system regulating phosphate starvation (*phoURB*) were frequently detected, mainly contributed by *Pseudomonadota*, *Gemmatimonadota* (*phoU*), and *Actinomycetota* and *Planctomycetota* (*phoR*). *PhoURB* showed a slight increase in PA over time, with *phoR* significantly more abundant in PA than SG.

Regarding organic P sources from plants or membrane phospholipids, *phn* phosphonate transporter genes (*phnCDE*) and sn-glycerol 3-phosphate (G3P) transporter genes (*UgpE*, *UgpC*, *UgpB*, and *UgpA*) were transcribed. The *glpQ* gene product cleaves these compounds, releasing G3P and activating *ugp* transporter genes. *Actinomycetota*, *Pseudomonadota*, *Deinococcota*, *Chloroflexota*, and *Bacillota* contributed to the transcription of these genes. In PA, *glpQ* and *UgpB* increased, while *UgpC* and *UgpA* decreased from T1 to T3, with no clear trends in SG. Moreover, PA had a significantly higher *phnD* and *glpQ* transcription abundance than SG. Lastly, the C–P lyase multienzyme complex genes (*phnGHIJLM*), responsible for acquiring P from phosphonates, were identified but not transcriptionally active.

#### Sulfur metabolism

For S metabolism, dissimilatory sulfate reduction (DSR), assimilatory sulfate reduction (ASR), and the sulfur-oxidizing system (SOX) were examined (Figs. 4, 5, Additional file 2: Table S2). Metatranscriptomic analysis revealed the absence of DSR pathway markers *dsrA* and *dsrB*, which are responsible for sulfite-to-sulfide conversion. In contrast, the ASR pathway dominated S metabolism, with all pathway genes active (*PAPSS*, *sat*, *cysND*, *cysC*, *cysH*, *cysJI*, and *sir*). While SG did not display clear change trends, in PA, *cysJ*, *cysC*, and *sat* decreased, whereas *sir* increased from T1 to T3. Moreover, *cysI* and *cysC* were significantly more abundant in PA than SG. *Actinomycetota* and *Pseudomonadota* were primary contributors to the ASR pathway, with *Planctomycetota*, *Bacillota*, *Bacteroidota*, *Chloroflexota*, and *Nitrososphaerota* also involved. Lastly, the SOX system was present in low abundance for thiosulfate to sulfate conversion, whose genes were (*soxA*, *soxYZ*, *soxB*, and *soxC*) carried by *Pseudomonadota*.

### Differentially expression levels due to site

**Fig. 6:**
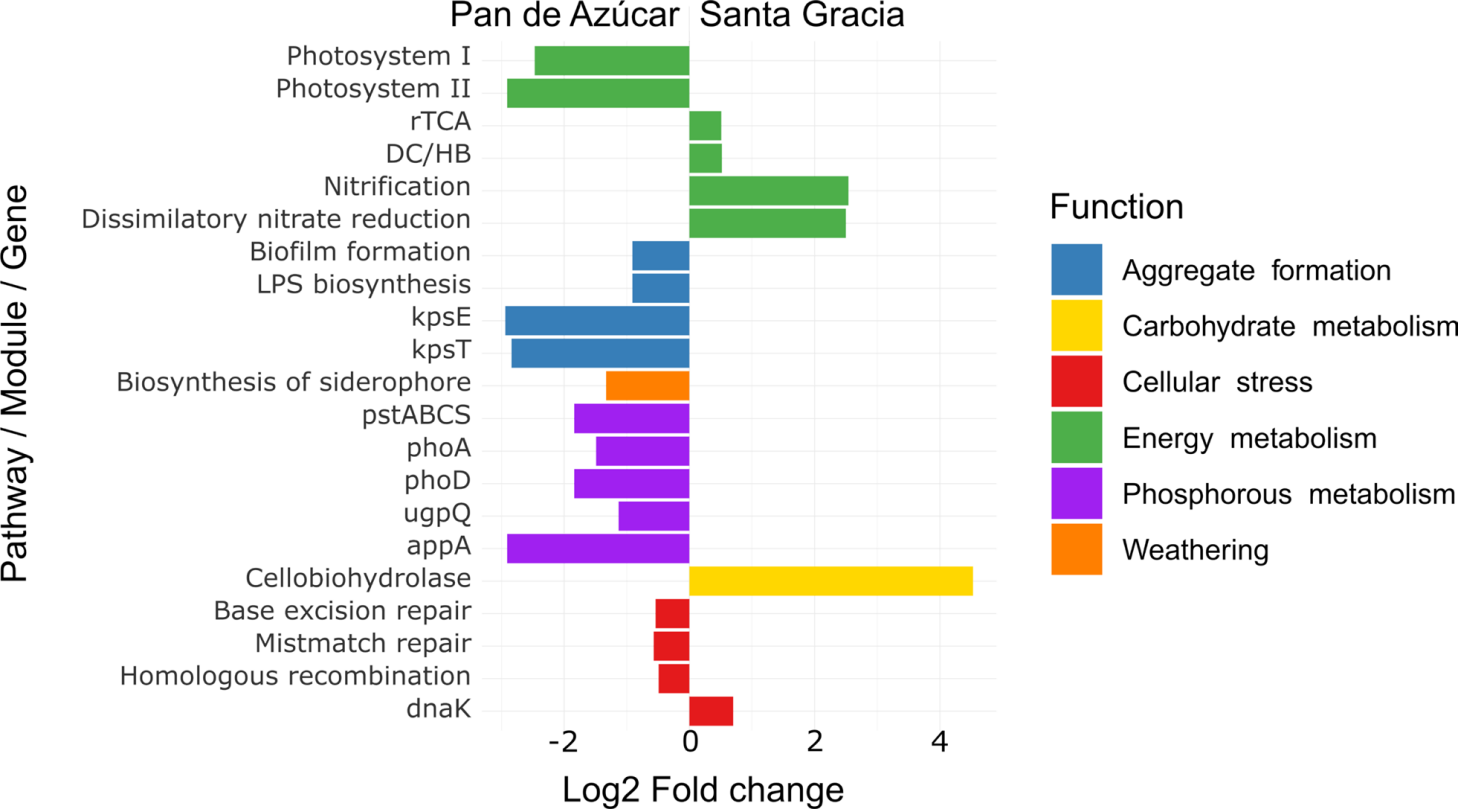
Differential expression analysis by DESeq2 compares the mean expression levels between Pan de Azúcar and Santa Gracia sites, incorporating different time points (2 weeks and 16 weeks) and treatments (in situ and plant) for each site. A negative fold change indicates significant enrichment of genes, modules, or pathways in Pan de Azúcar samples, while a positive fold change indicates significant enrichment in Santa Gracia samples. Detailed statistical results for all analyzed genes can be found in Additional file 2: Table S9

DESeq2 analysis revealed significant differences in KEGG orthologs expression between sites (*p-value* < 0.05, Fig. 6, Additional file 2: Table S9). The enrichment analysis of statistically significant genes highlighted distinct functional trends: PA showed significant enrichment in EPS (biofilm formation, LPS biosynthesis, *KpsT*, and *KpsE*), P-metabolism (*phoA*, *phoD*, *upgQ*, *appA*, and *pstSABC*), siderophore biosynthesis, energy metabolism (Photosystem I and II, *rbcL*, *rbcS*, *prk*), and cellular stress (base excision repair, mismatch repair, homologous recombination, and *dnaK*). In contrast, SG exhibited significant enrichment in energy metabolism (rTCA, DC/HB, nitrification, DNR) and, to a lesser extent, in carbohydrate metabolism (*CBH2, abfA*).

## Discussion

Microbial activity is pivotal in early soil formation in nutrient-poor arid and semiarid soils with sparse vegetation [42, 59]. It utilizes electrons from bedrock for energy, produces biofilm matrices, and mobilizes nutrients [60]. However, limited precipitation makes this process slow in arid and semiarid sites. Therefore, stimulating these soils with controlled humid climate conditions is crucial to understanding how soil microbial composition and function drive soil formation dynamics in these challenging environments and provide hints on how initial soils can develop under a climate change scenario.

### Microbial-driven weathering capacity increases in soils with lower bioavailable P under humid conditions

Microbial activity in PA soils exhibited enhanced expression of weathering-related genes under humid conditions, especially the *gcd* gene, which is responsible for the biosynthesis of organic acid anions, such as gluconic and 2-keto gluconic acid [16, 20]. This upregulation indicates an increased microbial capacity for solubilizing mineral-P from apatite in PA by the end of the experiment, potentially accelerating P release from the bedrock to support living organisms. In contrast, the SG site showed no variation in *gcd* gene abundance over time, likely due to higher bioavailable P in these soils. Our findings support studies indicating that increased precipitation enhances apatite dissolution, leading to higher plant-available P from PA to SG [24, 61]. Furthermore, the released organic acids likely decreased soil pH in PA along the simulation, consistent with observations in Damma glacier forefields [42].* Pseudomonadota*, particularly *Alphaproteobacteria*, increased over time and are the main contributors to the *gcd* gene abundance, consistent with prior research identifying them as key players in P solubilization [61, 62]. These findings underscore the potential acceleration of microbial P solubilization in arid sites under humid conditions by releasing organic acids in soils with lower bioavailable P, enhancing weathering.

We did not detect active genes responsible for Fe solubilization, like iron:rusticyanin reductase, which is involved in soil development via ferrous Fe oxidation [63]. However, we observed increased transcription of the *fhuE* gene and active siderophore biosynthesis, which are crucial in Fe uptake [64] and the chelation of Fe^3+^ on the cell membrane, facilitating Fe solubilization [60]. This process might be critical in Fe-deficient PA [24], where the pathway showed higher transcription levels than in SG. Our findings indicate that P and Fe demand by microorganisms may boost weathering and soil formation in arid soils when the climate becomes more humid, especially when these nutrients are scarce, such as in PA compared to SG, due to their critical role in biological functions.

### Microorganisms enhance EPS metabolism as a potential adaptative response

In addition to weathering, the increased gene expression for EPS synthesis and transport under humid conditions suggests that microbes promote microaggregate formation in PA by adhering to soil particles and connecting them into larger structures. We observed an upregulation of the biofilm formation pathway and specific EPS synthesis genes (*kpsE* and *kpsT*), correlating with increased microaggregation. Similarly, the gradual abundance increase of the export polysaccharides *wza* gene suggests improved soil structure stabilization, as observed in studies comparing biocrusts or alfalfa-planted soils with bare soils [23, 65]. This increase is complemented by a rise in *exoP*, a polysaccharide transport protein linked to bacterial survival in stressful conditions through EPS [66]. Active gene transcription for colanic acid biosynthesis (*wcaI*, *wcaK*, or *wcaL*) was also detected, potentially linked to their prevalent role as EPS in soils [22]. The upregulated genes were carried mainly by *Pseudomonadota*, especially *Sphingomonadaceae*, consistent with findings during the initial reclamation of mining areas and nutrient-poor tilled soils [23, 66]. Similar to reports linking EPS abundance to stress responses in disturbed environments such as tillage or cultivation [22, 23], our results highlight the enhancement of EPS-metabolism genes under altered climate conditions in PA, which can act as stressors, potentially promoting soil aggregation.

The upregulation of EPS-metabolism genes in PA may be a response to particle size and stress conditions. Higher clay content promotes efficient soil aggregation through physicochemical forces and enhanced production of EPS by microorganisms [67, 68]. Consequently, clayey soils of PA could promote soil stabilization and protection of bacterial communities within the aggregates [24, 66]. Additionally, microorganisms produce EPS and biofilms to defend against environmental stresses, predation, and phagocytosis [22, 69]. Therefore, increased EPSs-metabolism also serves as a defense mechanism against predators like *Myxococcota* [70, 71], which exhibited higher transcriptional activity following humid conditions. These findings underscore how humid conditions may enhance bacterial-mediated soil stabilization through EPS, potentially improving bacterial resilience in PA soils.

The microbial resilience and lower clay content in SG may limit the competitive advantage of EPS. SG showed decreasing microaggregates and increasing macroaggregates, with stable EPS-metabolism gene abundances, likely due to its generalist community adapted to environmental fluctuations [6]. These soils reduce reliance on costly EPS matrices, as they rely less on bacterial biomass for aggregation due to their coarser texture [72], favoring macroaggregation facilitated by fungi with mycelial hyphae [60]. However, bacterial activities degrading organic compounds into C sources may support fungal growth and mycelial development [60], indirectly enhancing macroaggregate stability. This potential enhancement could be crucial in macroaggregate formation, even without increased EPS production by bacteria and archaea.

### Adaptations of the microbial community to environmental changes alter the C, N, and P dynamics of the soil

The shifts in microbial activity associated with C, N, and P dynamics highlight the adaptability of soil microorganisms to environmental changes. Reduced or absent transcription of genes involved in autotrophic C and N fixation, coupled with upregulation of genes related to P and organic substrate utilization, suggests a strategic metabolic reconfiguration linked to soil nutrient dynamics. These adjustments likely optimize organic resource utilization by heterotrophs in response to increased moisture availability [35], facilitated by organic compound release from microbial death via osmolysis and organic matter turnover [73, 74]. Furthermore, soil communities may rely on C and N inputs from chemoautotrophs and alternative sources such as sporadic vegetation growth, hypoliths, or biocrusts [31, 35]. These adaptive changes are crucial in shaping soil nutrient availability and aggregation dynamics during initial soil development stages.

N-fixation gene transcriptions are absent in PA and SG under humid conditions, with minimal presence in the metagenome. Contrary to previous findings that identified *Cyanobacteria* and archaea as primary contributors [60], *Pseudomonadota* and *Bacillota* were predominant. These findings support studies in Tarim Basin soils and halites of Atacama and Namib Deserts, which also showed limited or no evidence of N-fixation [16, 19, 75, 76]. However, a slight increase in these gene abundances in the metagenome suggests potential induction of anoxic conditions due to increased moisture, hinting at possible N-fixation in longer-term experiments. These bacteria require anaerobic or microaerophilic environments [77].

Under humid conditions, soil bioavailable N decreases due to leaching in sandy soil in PA and microbial activity in SG. Despite essential genes for ANR, DNR, and denitrification in the PA metagenome, their transcription remains absent, consistent with low denitrification rates in other desert soils despite high NO_3_^−^ levels [16]. Moreover, potential inhibition of denitrifying microbes in coastal soils sensitive to salinity shifts from freshwater application may occur [60, 78]. Although denitrification, ANR, and DNR are possibly inactive, and nitrification potentially increased due to moisture, NO_3_^−^ loss in PA by the end of the experiment is likely due to leaching. In contrast, denitrification was active in SG, with the *nosZ*, *norBC*, or *napA* genes correlating with soil NO_3_^−^ content. This process, potentially driven by labile C after rewetting, is favored in nitrate-limited soils with C-content, supporting denitrification activity in SG over PA, as observed in pasture soils [79]. Additionally, nitrification genes showed lower rhizosphere transcription than in situ soil and a slight downregulation over time, consistent with studies in arid ecosystems post-precipitation [80]. As previously highlighted, *Pseudomonadota* and ammonia-oxidizing archaea *Nitrososphaerota* play significant roles in nitrification [19, 77]. These observations suggest that limited microbial N metabolism may restrict soil formation, impacting nutrient availability and soil water retention, which are critical for soil fertility and ecosystem function in PA. Meanwhile, initial microbial N metabolism activity in SG decreases over time, possibly due to adaptation to favorable conditions and sufficient nutrient availability from organic matter.

C metabolism undergoes restructuring in PA, potentially influenced by oxygen availability. Initially, the Calvin cycle, crucial for microbial C-fixation in drylands [16, 19], exhibited heightened expression but declined over time. This reduction is likely due to oxygen-sensitive enzymes, such as RubisCO (*rbcL* and *rbcS*) and glyceraldehyde-3-phosphate dehydrogenase [18, 30, 80]. Increased soil moisture to 65% water-filled pore space induces anoxic conditions in micropores. Assuming that the micropores proportion is higher in clayey soils than in sandy soils [81], anaerobic pathways are favored in clay-rich PA soils. Enzymes in H_2_ oxidation (*hhyl* and *hhys*), supporting aerobic respiration under low C availability, as in the Calvin cycle [31, 32], also decrease over time. Conversely, the rTCA cycle was the predominant pathway for synthesizing C compounds from CO_2_ and water in PA and SG under humid conditions. Both sites exhibited a substantial capacity for chemosynthetic C-fixation, as seen in studies of desert halites, biocrusts, and arid soils [19, 31, 82]. Key enzymes like *korA* and *korB* increased remarkably from T1 to T3, similar to findings in irrigated semiarid sites [30]. Additionally, the rTCA and DC/HB pathways, utilized by anaerobic or microaerophilic microbes, demand less energy than the aerobic Calvin cycle [18]. *Actinomycetota* were the major contributors to the Calvin cycle, as in Antarctic desert soils [83]. The transition from the Calvin cycle to the rTCA cycle as the dominant pathway in PA is accompanied by a shift in microbial taxa, with a decrease in *Actinomycetota* and an increase in associated *Pseudomonadota*. Our finding indicates that chemosynthesis replaces photosynthesis for C cycling, reflecting an evolutionary trajectory of soil microbes favoring low ATP requirements pathways dependent on oxygen availability rather than being optimized for current environmental conditions.

Microbial communities in SG and PA utilize organic matter degradation alongside N and C fixation. Under humid conditions, increased enzymatic activity can release protected organic compounds and osmolytes. In SG, *CBH2* (cellulose degradation) was upregulated at T1, particularly in plant treatments, correlating with TOC and N_bulk_, as observed in studies on straw amendments and dry-wetting cycles [74, 84]. Similarly, xylanase and pectate-lyase, targeting plant cell wall polysaccharides, were upregulated at T1 in SG, which coincides with the peak of enzymatic activity assays observed. In PA, *lacZ*, *FUCA* (plant cell wall degradation), and chitinase (fungal cell wall degradation) were upregulated at T3, with chitinase showing high levels in plant treatments. This supports findings of higher transcription in agricultural compared to arid sites [85]. Additionally, chitinase correlated with *wza*, *kpsE*, and *kpsT*, suggesting the utilization of substrates such as chitin for EPS synthesis. As shown in previous studies and supported here, *Actinomycetota* are key degraders of complex carbohydrates like hemicelluloses and xylan [16]. This enzymatic upregulation, particularly in response to plant treatments, underscores the role of microorganisms in plant decomposition, soil organic matter renewal, and promoting soil structure improvement.

Gene upregulation related to inorganic and organic P uptake and transport occurred in PA under humid conditions, highlighting microbial challenges in P utilization in nutrient-poor desert environments. In PA, efficient inorganic phosphate transporters, such as *pst* transporter subunits and the *pit* system (transporting metallic cations complexed with P), exhibited higher transcriptional abundance than SG. These transporters are critical in P-depleted soils, enabling microorganisms to compete with plants for bioavailable P [17, 86]. Genes involved in the two-component system (*phoB* and *phoU*), crucial during P starvation [16, 86], were more abundant in PA than SG, emphasizing efficient gene regulation and facilitating alternative P source utilization. Additionally, microbes in PA potentially use phosphonates as an organic alternative P source. These can be degraded by alkaline phosphatases *phoA* and *phoD*, which target monoester bonds and broader substrates such as teichoic acids and phospholipids [16, 86]. Enzymes like *glpQ*, *ugpB*, and *appA* indicate the release and transport of G3P from plant exudates and phospholipids [17]. High *appA* gene abundance in PA indicates microbial utilization of phytate from plant storage, demonstrating proficiency with various P sources. Conversely, SG exhibited lower transcription levels of genes involved in P metabolism, likely due to higher bioavailable P [24]. *Pseudomonadota* and *Actinomycetota* were observed to play roles in P cycling processes, as found in other desert and forest soils [16, 86]. The increased expression of P metabolism-related genes under humid conditions highlights efficient P control mechanisms in PA, which are crucial for overcoming limitations in oligotrophic environments with low organic matter.

The microbiome in both soils actively transcribes genes for ASR, potentially influenced by sulfate availability transported via coastal fog [75] and correlates with reduced soil sulfate content. Therefore, S assimilation likely contributes to nutrient cycling and soil stabilization by converting S into organic forms. However, DSR genes were absent in both sites, consistent with findings in Namib desert soils [75], indicating a requirement for anoxic conditions.

### Soil formation processes are driven by soil legacy and treatments

PA exhibited higher differentiation in metagenomic and metatranscriptomic profiles compared to SG under humid conditions, indicating a higher specialization and sensitivity to environmental disturbances in the microbial community of PA. This was already suggested through amplicon-based microbial community analyses at the same sites [39] and can now be substantiated. Rapid microbial responses in PA to disturbance confer adaptive advantages, activating dispersal mechanisms such as natatory (flagella) and gliding (type IV pili). These mechanisms help to explore new ecological niches and substrates, exploit moisture, and overcome stress [35]. Microorganisms in PA consistently displayed higher transcriptomic abundance of stress-tolerant genes involved in genetic information processing pathways like homologous recombination, base excision repair, and mismatch repair, supporting findings on bare soils [15]. However, humid conditions reduced cellular stress, as indicated by the down-regulation of stress-resistance genes (*otsB*, *HSP20*, *groEL*, and *dnaK*), associated with the decreased abundance of *Actinomycetota* and *Gemmatimonadota*. These shifts likely contributed to reduced cellular stress within the emerging microbial community, reflecting the historical legacy of desert soil microorganisms and revealing contrasting functional patterns driven by specific microbial requirements.

Taxonomic similarities between plant and in situ treatments (indicated by PCoA) show that climate factors predominantly influenced microbial community dynamics over specific treatments in the short term. However, transcriptional profiles of SG showed greater differences in in situ than in plant treatments, especially in pathways like N metabolism. This points to stable microbial functionality facilitated by plant interactions, influenced by pH, soil structure, oxygen availability, and carbon-rich exudates [87]. Therefore, plant-microorganism interactions seem crucial in stabilizing microbial community functionality, positively influencing soil structure improvement and nutrient availability.

## Conclusion

Soil formation is substantially influenced by changes in microbial activity in response to changing environmental conditions (e.g., metabolic needs and environmental stress). In soils from PA, climate change from arid to humid conditions triggers a rapid microbial response. Here, microorganisms appear to increase organic matter degradation and P solubilization, weathering, and EPS production, which promotes soil development and stabilization. In contrast, SG, with a less erratic precipitation pattern and slightly higher nutrient availability, exhibits a more stable functional core microbiota characterized by a less pronounced response to higher moisture. This difference in microbial activity between PA and SG reflects microbial adaptation to specific soil conditions, demonstrating how the interplay between microorganisms and the environment influences soil dynamics, development, and functionality, particularly under changing environmental conditions.

## Supplementary Information

Additional file 1.

Additional file 2.

## Data Availability

The DNA and RNA raw datasets presented in this study are available online in the ENA repository under the Project Accession Number PRJEB76434.

## Abbreviations

PA: Pan de Azúcar
SG: Santa Gracia
C: Carbon
P: Phosphorous
S: Sulfur
N: Nitrogen
EPS: Exopolysaccharide
LPS: Lipopolysaccharide
H_2_: Hydrogen
C_t_: Total Carbon
N_t_: Total Nitrogen
MWD: Mean weight diameter
TOC: Total organic carbon
N_bulk_: Total nitrogen in the bulk soil
EC: Electrical conductivity
KOs: KEGG orthology IDs
