# Supplementary material for: Microbial transcriptome patterns highlight increased pedogenesis-related activity in arid soils under simulated humid conditions
Source: Environ Microbiome. 2025 Mar 17;20:31. doi: 10.1186/s40793-025-00689-3 (PMC11917034; doi:10.1186/s40793-025-00689-3)
Supplement: Supplementary file 1 — Additional file 1. [file 40793_2025_689_MOESM1_ESM.docx]

**Material supplementary**

**Microbial transcriptome patterns highlight increased pedogenesis-related activity in arid compared to semiarid soils under simulated humid conditions.**

Victoria Rodríguez, Alexander Bartholomäus, Susanne Liebner, Romulo Oses, Thomas Scholten, and Dirk Wagner


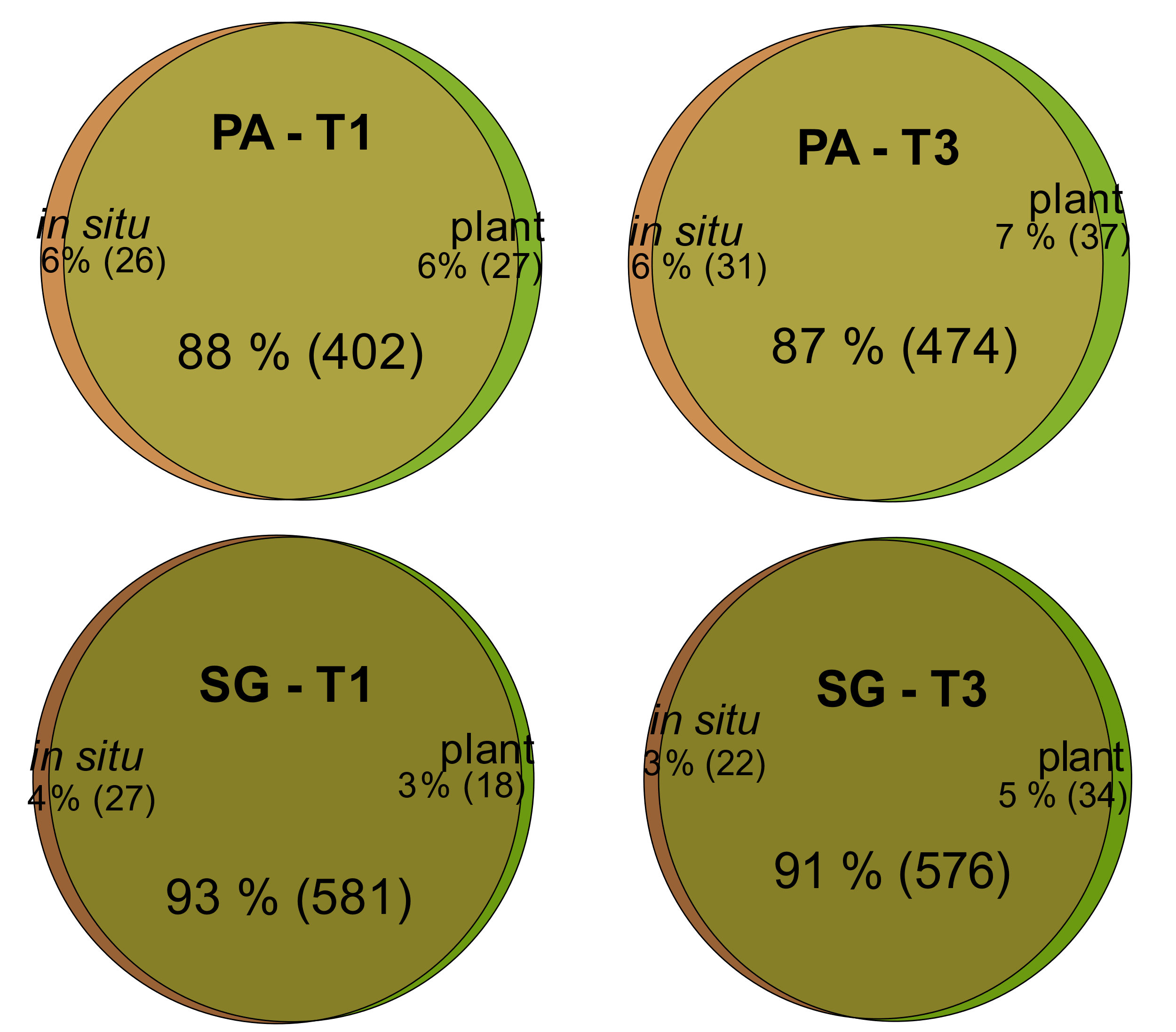


**Fig. S1.** Venn diagrams show the number of shared and unique ASVs among the two treatments (*in situ* and plant) over time (T1 and T3) for Pan de Azúcar (PA) and Santa Gracia (SG). The shared ASVs overlap the brown and green circles. The diagrams show the ASVs that summed over 1% relative abundance for all samples. The data is available in Rodriguez et al. (2024).


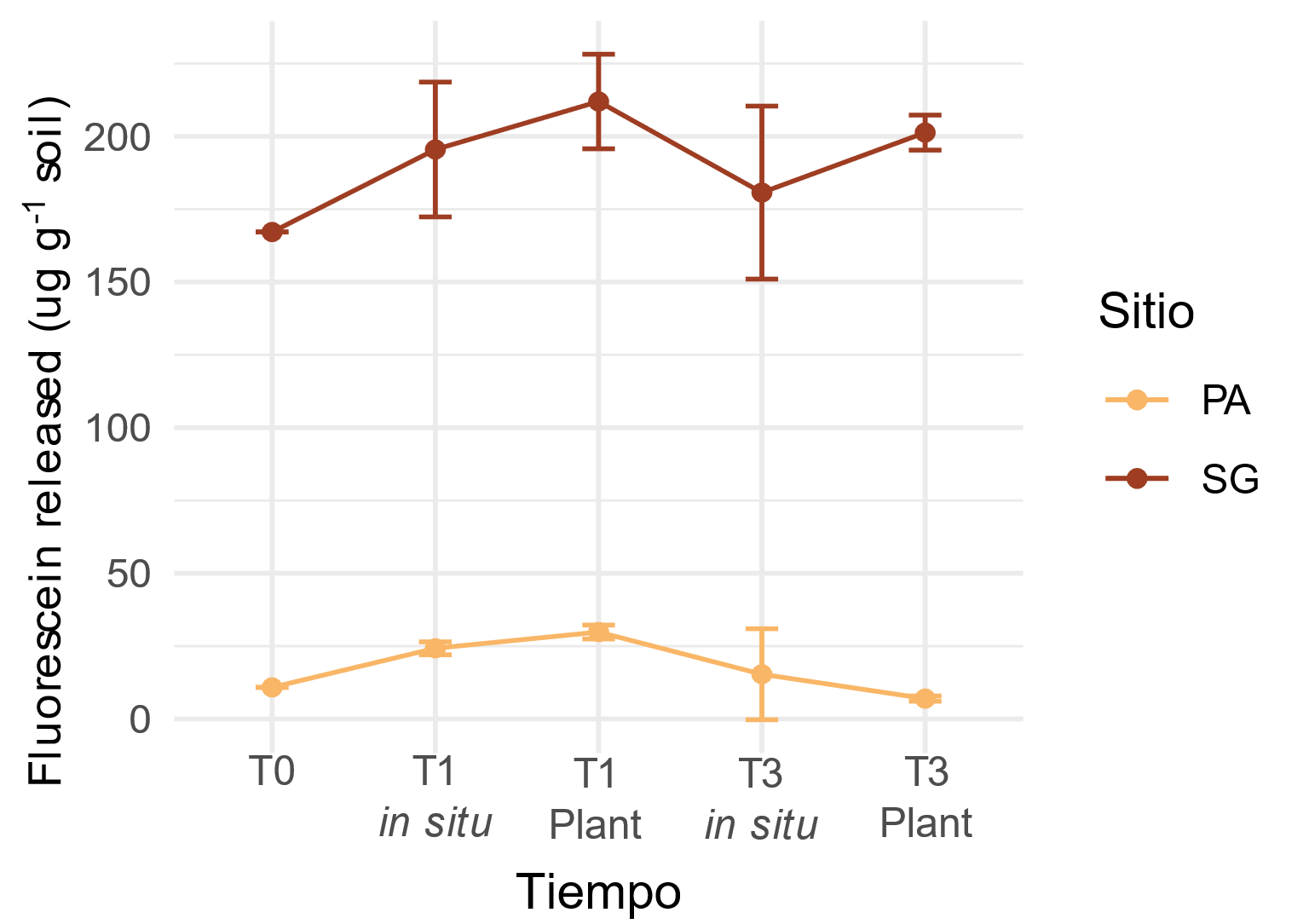


**Fig. S2.** Release of fluorescein during FDA hydrolysis in Pan de Azúcar (PA) and Santa Gracia (SG). Points represent the mean of three replicates; error bars represent the standard deviation.


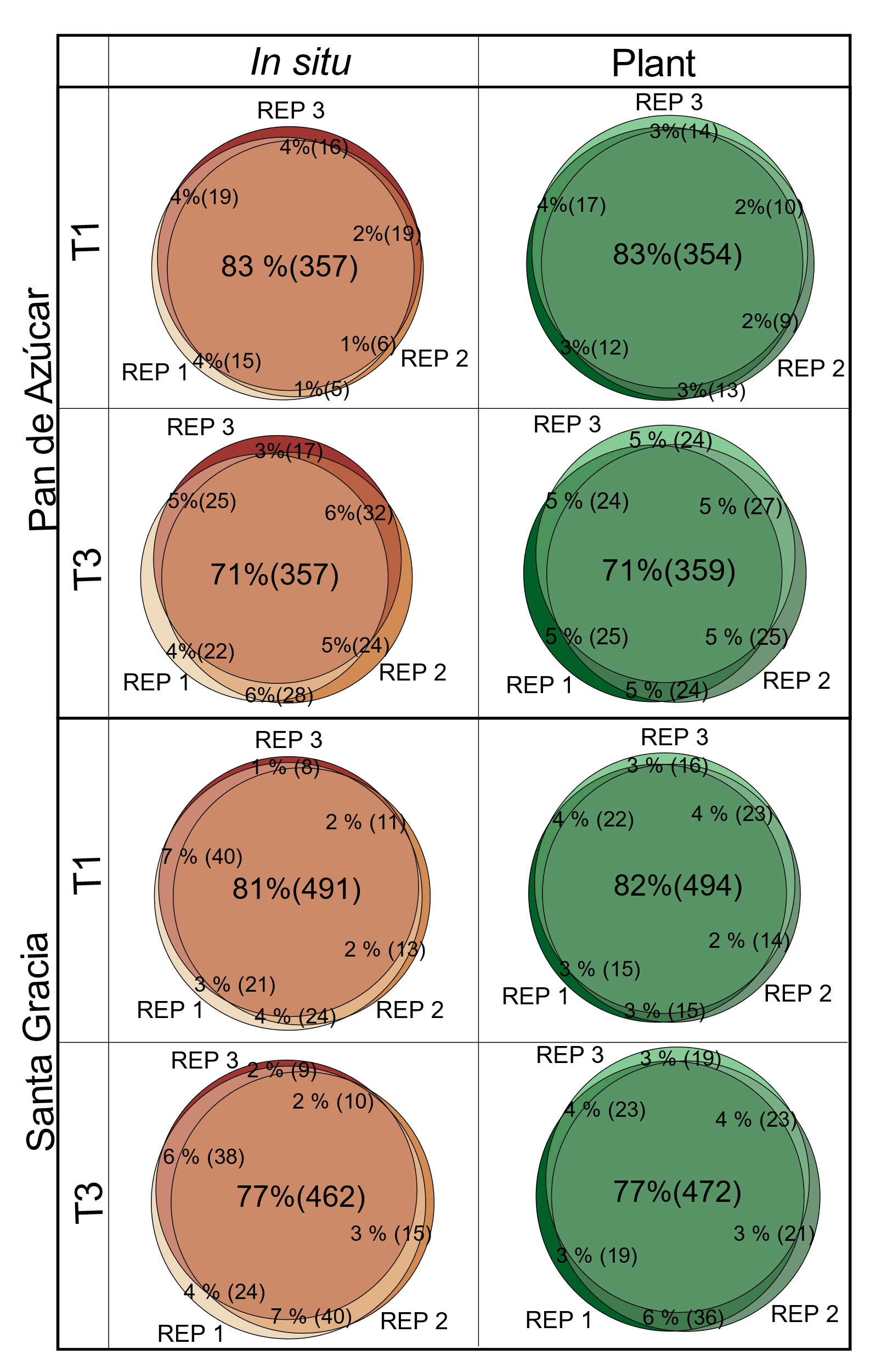


**Fig. S3.** Venn diagrams show the number of shared and unique ASVs among the three biological replicates. The diagrams are separated by treatments (*in situ* and plant) over time (T1 and T3) for Pan de Azúcar and Santa Gracia. The overlapping ASVs are present across all three distinct replicates. The diagrams show the ASVs that summed over 1% relative abundance for all samples. The data is available in Rodriguez et al. (2024).


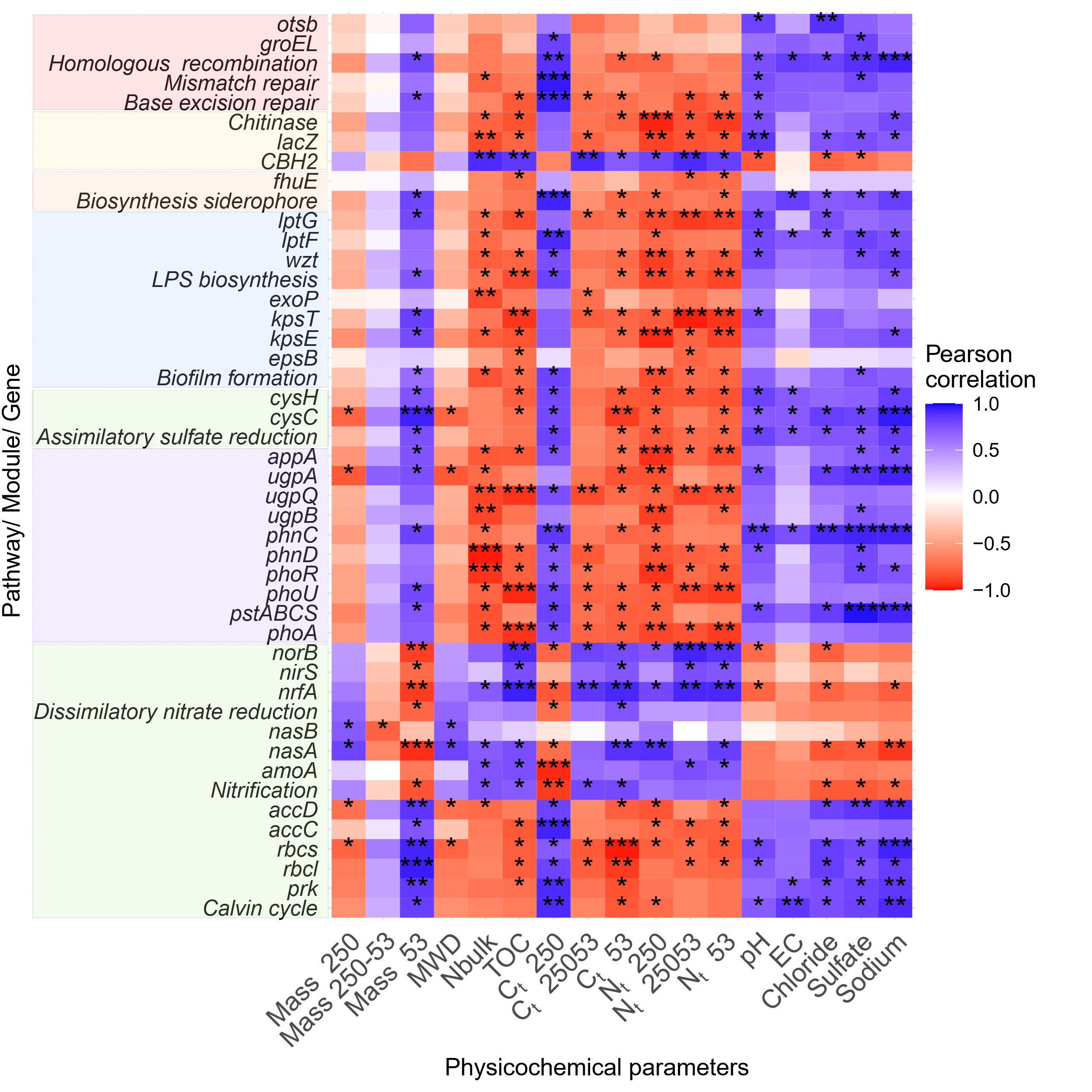


**Fig. S4.** Correlation between the gene expression levels of pathways, modules, or genes and soil physicochemical properties evaluated in Pan de Azúcar and Santa Gracia soils. Color boxes represent the metabolism, where red represents cellular stress, yellow represents carbohydrate metabolism, orange represents weathering, blue represents aggregate formation, purple represents phosphorous metabolism, and green represents energy metabolism. The physicochemical properties included total mass in macroaggregates (Mass 250), total mass in large microaggregates (Mass 250–53), total mass in small microaggregates (Mass 53), mean weight diameter (MWD), nitrogen in the bulk soil (N_bulk_), total organic carbon (TOC), total carbon in macroaggregates (C_t_ 250), total carbon in large microaggregates (C_t_ 250–53), total carbon in small microaggregates (C_t_ 53), nitrogen in macroaggregates (N_t_ 250), nitrogen in large microaggregates (N_t_ 250–53), nitrogen in small microaggregates (N_t_ 53), pH, electrical conductivity (EC), and sodium, chloride, and sulfate concentration.
